# Medical image segmentation of gastric adenocarcinoma based on dense connection of residuals

**DOI:** 10.1002/acm2.14233

**Published:** 2023-12-14

**Authors:** Ying Hu, Yue Guo, Xian Xu, Shipeng Xie

**Affiliations:** ^1^ College of Communications and Information Engineering Nanjing University of Posts and Telecommunications Nanjing China

**Keywords:** 3D medical image segmentation, CT, gastric adenocarcinoma, residual dense skip block

## Abstract

**Background and objective:**

Accurate segmentation of gastric cancer based on CT images of gastric adenocarcinoma is crucial for physicians to screen gastric diseases, clinical diagnosis, preoperative prediction, and postoperative evaluation plans. To address the issue of the inability of the segmentation algorithm to depict the correct boundaries due to unclear gastric contours in the lesion area and the visible irregular band‐like dense shadow extending to the perigastric region, a 3D medical image segmentation model 3D UNet based on residual dense jumping method is proposed.

**Methods:**

In the method we proposed, Residual Dense Block, which is applied to the image super‐resolution module to remove CT artifacts, and Residual Block in ResNet are further fused. The quality of CT images is improved by Residual Dense Skip Block, which removes banded dense shadows, preserves image details and edge information, captures features, and improves the segmentation performance of gastric adenocarcinoma. The Instance Normalization layer position is modified to select the best result. Different loss functions are also combined in order to obtain the best gastric adenocarcinoma segmentation performance.

**Results:**

We tested the model on a hospital‐provided gastric adenocarcinoma dataset. The experimental results show that our model outperforms the existing methods in CT gastric adenocarcinoma segmentation, in which the method combining the hybrid loss function of Dice and CE obtains an average dice score of 82.3%, which is improved by 5.3% and 3.8% compared to TransUNet and Hiformer, respectively, and improves the cross‐merge rate to 70.8%, compared to nnFormer, nnUNet by 1% and 0.9%, respectively.

**Conclusions:**

The residual jump connection structure indeed improves segmentation performance. The proposed method has the potential to be used as a screen for gastric diseases and to assist physicians in diagnosis.

## 1 INTRODUCTION

With the rapid development of the economy and the increasing improvement of living standards, people are more and more concerned about health issues. Cancer has become one of the major diseases that jeopardize human health. According to the 2018 Global Cancer Incidence and Mortality Statistics Report, there were about 1.034 million new patients with gastric cancer and as many as 783,000 deaths from gastric cancer globally in 2018, a figure much higher than the world average.^1^ The emergence of computed tomography image technology (CT) has become an important tool and method for early screening, examination, and diagnosis of gastric cancer patients.

Enhanced CT of the upper abdomen plays an important role in segmenting gastric cancer, determining resectability and the extent of surgical resection, and selecting treatment options. In this case, the site of the disease was the cardia fundus area, and the analysis of gastric cancer based on CT images is a challenging task in itself. CT images can show irregular strips and bands of dense shadows in the stomach with unclear contours, rough plasma membrane surfaces, and the lesion area stretching out to the periphery of the stomach. At the same time, the shape and size vary greatly from patient to patient, intensity similarity with non‐stomach tissues and smooth or invisible borders (due to resolution limitations of medical scanners) are issues that need to be addressed in a successful segmentation approach.

In recent years deep learning has been effectively applied and well recognized in medical image analysis of many body parts, such as medical image segmentation,^2^ lung nodule detection and classification,^3^ and medical image super‐resolution.^4^ In medical image segmentation, there have been numerous scholars proposing different network models, mostly focusing on network structure improvement to enhance segmentation accuracy. Özg¨unC¸i¸cek et al.^5^ proposed a method to learn dense group segmentation from sparse annotations, that is, 3D‐UNet, where three‐dimensional images do not need to be input into each slice individually for training, but instead, the whole of the picture can be taken as input to the model. Milletari et al.^6^ proposed a 3D image segmentation network based on a fully convolutional neural network design, that is, V‐Net. the model introduces horizontal residual connectivity as well as adopts 3D convolution technique to avoid losing too much edge and detail information by downsampling or upsampling. Also, the proposed loss function (Dice Loss) can deal with the imbalance in the number of foreground and background pixels, making it more suitable for 3D medical image segmentation. X. Li et al.^7^ proposed a novel hybrid densely connected UNet model (H‐DenseUNet), which fuses the advantages of densely connected paths and UNet connections. Gibson et al.^8^ fused dense connections with V‐Net and used dense connections in each convolutional layer of the original V‐Net for simultaneous segmentation of multiple organs in the abdomen. Tomoyuki et al^9^ achieved early gastric cancer segmentation by analyzing and detecting the gastrointestinal endoscopic images, but it was based on the target detection method, and the segmentation accuracy was not very high. Sun et al.^10^ implemented gastric cancer segmentation in pathological images using variable convolution and multi‐scale networks, with a segmentation accuracy of 82.65% on pathological images. Xiao et al^11^ and Wang et al^12^ replace the convolution module in UNet with residual (ResNet) idea and dense connectivity (DenseNet) idea, respectively, to realize ResUNet for segmentation of retina image and DenseUNet for denoising of the image and both of them achieve very good. In 2018 Fabian Isensee et al.^13^ proposed nnUNet, an adaptive medical image segmentation framework based on U‐Net, which focuses on preprocessing, training, inference, and post‐processing, and achieves superior performance in 10 different medical image segmentation tasks. nnUNet does not propose a new structure and relies on a few tricks to make the segmentation task more unified. The authors believe that more improvement actually lies in understanding the data and adopting appropriate preprocessing and training methods for medical data. In some cases, the more the structure is modified, the easier it is to overfit.

Therefore, we propose a 3D medical image segmentation method based on residual jump structure for common gastric adenocarcinoma from CT image quality improvement, in order to provide auxiliary references for physicians to make clinical diagnosis. From, ref.^14^, ^15^, ^16^ we know that the structure of residual network shows good performance in removing streak artifacts, while the residual unit simplifies the training of deep networks while the rich jump connections can promote information propagation, allowing the design of networks with fewer parameters and better performance. Inspired by the work of,^17^, ^18^, ^19^ the residual dense block proposed by RDN^18^ for CT image reconstruction and stripe artifact removal was fused with the residual block proposed by ResNet.^17^ A residual dense skip block suitable for medical image segmentation was proposed, which was combined with 3D UNet to construct an end‐to‐end encoder‐decoder structure for 3D medical image segmentation models. Residual dense skip blocks (RDSB) can effectively extract targets of different scales and their edges, and can more effectively segment CT images of gastric adenocarcinoma with complex conditions, demonstrating good performance in image feature extraction. The experimental results show that the model proposed in this paper outperforms algorithms such as HiFormer, TransUNet, and nnUNet in Dice, IoU, and HD95. The segmentation results obtained in terms of 3D segmentation of gastric adenocarcinoma disease area are more accurate and the best current performance of gastric adenocarcinoma segmentation is obtained.

## 2 METHODS

**FIGURE 1:**
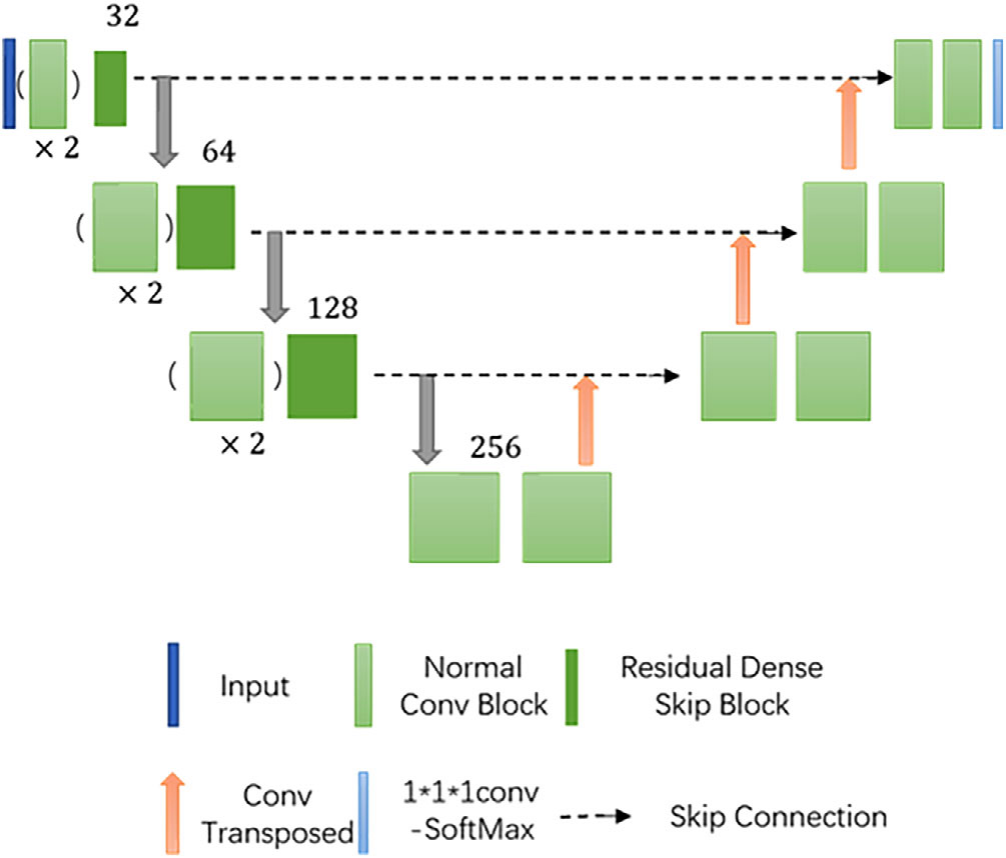
Improved network module.

The skip connection structure can deepen the network depth and improve the network performance, but at the same time, due to the original dataset format of Dicom, blurring and artifacts of CT images are inevitable. By studying the unified medical image segmentation framework of nnUNet, we learned that more attention should be paid to the pre‐processing of the dataset. By improving the data image quality, we are able to preserve image details and edge information, further capture features, and improve segmentation performance. Therefore, we consider the further fusion of RDB, which is applied to the image super‐resolution module, and RB, which is used in ResNet,^17^ to improve it. By improving the quality of CT images and obtaining more accurate feature details, we aim to improve the segmentation performance. Experimentally, our method proved to be superior in gastric adenocarcinoma segmentation, and the edge segmentation performance was further improved. Figure 1 shows the proposed 3D UNet model based on residual skip structure.

The basic composition of Normal Conv Block is Conv, Instance Normalization, Leaky ReLU, where kernal size is 3× 3× 3 and stride is 2, × 2 denotes that a block is repeated two times.

Residual Dense Skip Block uses dense skip connection, and subsequently, to improve the computational speed, the 3× 3× 3 convolution is changed to 3× 3× 1 and 3× 1× 3, and the stride is 1. Initial number of channels is 32.

### 2.1 Residual network

Convolutional neural networks often consist of a convolutional layer, a pooling layer, an activation function, and a fully connected layer. Improving the network's ability to extract image features can often be done by stacking convolutional layers to increase the network depth. However, as the convolutional neural network gradually deepens, the network model becomes more and more difficult to train, and the network performance starts to degrade and cannot achieve the expected results. ResNet^17^ further deepens the network depth by introducing a skip connection structure, and the gradient disappearance problem is further solved and the network performance is improved.

**FIGURE 2:**
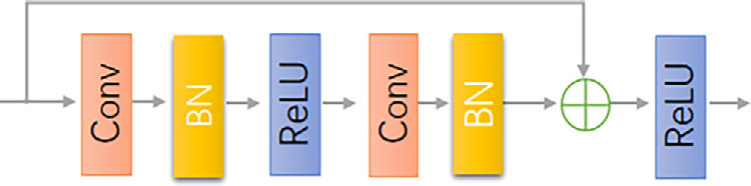
Residual block.

Figure 2 shows the basic residual blocks that form ResNet18 and ResNet34, consisting of residual paths and constant connection paths. The residual path consists of a Batch Normalization (BN), a 3 × 3 convolutional layer, and a ReLU activation function, and then the results of the two paths are summed by the ReLU to be the output. At the same time, the use of skip connections does not introduce redundant parametric quantities and the computational complexity remains unchanged.

**FIGURE 3:**
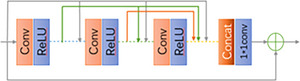
Residual dense block.

The residual dense block (RDB) proposed by RDN^18^ can further improve the network performance by extracting multi‐level features from the original image, as shown in Figure 3. RDB is done by local dense connectivity in order to fully utilize all the layers in it, and then the accumulated features are preserved adaptively by local feature fusion (LFF), while global residual learning is used in order to obtain global dense features, using global residual Learning combines shallow and deep features together for global feature fusion.

RDN introduces dense connections and dense blocks^20^ compared to the EDSR^21^ stacking building block. The key to the progress of RDN in deep neural networks is the addition of additional skip connections between different levels of features. Experimental results demonstrate that dense connections can improve the performance of image reconstruction networks, so we think about applying dense connections for image reconstruction to image segmentation and expect good results.

### 2.2 Residual dense skip block architecture

**FIGURE 4:**
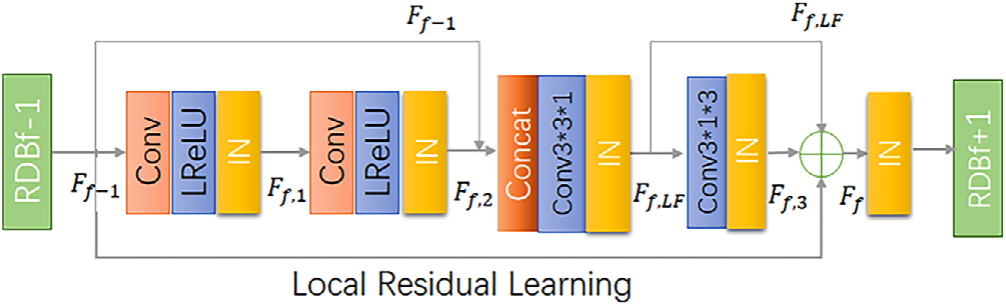
Residual dense skip block.

We propose the residual dense skip block (RDSB) for medical image segmentation of gastric adenocarcinoma, as shown in Figure 4. Many experiments have demonstrated that batch normalization is suitable for segmentation of medical images. However, here, we introduce Instance Normalization (IN) in RDSB and demonstrate that the segmentation accuracy obtained by adding IN layer effect is better, and the ReLU function is replaced by Leaky ReLU function.

#### 2.2.1 Residual dense skip block

In Figure 4, we introduce the output of the f−1th RDB with $F_{f,2}$ for feature fusion, on the other hand, inspired by the RDN,^18^ we replace the size of the convolutional layer to adaptively control the output information. The local feature fusion equation can be expressed as:

(1) $$F_{f,LF}=C_{LFF}^{f}\;\left[F_{f-1},F_{f,2}\right]$$

where the fth RDSB local feature fusion result is denoted by $F_{f,LF}$. $C_{LFF}^{f}$ denotes the concat feature map stacked convolution normalization function in thefthRDSB. *F*_2_ denotes the result of shallow feature extraction by the fth RDSB input after two convolutional layers, two IN layers, and LReLU activation function, and $F_{f,2}$ can be expressed as:

(2) $$F_{f,1}=\;IN\left[\delta \left(W_{f,1}⊗F_{f-1}+b_{1}\right)\right]$$

(3) $$F_{f,2}=\;IN\left[\delta \left(W_{f,2}⊗F_{f,1}+b_{2}\right)\right]$$

where, δ denotes the ReLU activation function, $W_{f,1}$, $W_{f,2}$ denotes the weights of the 2nd convolutional layer and the 1st convolutional layer, respectively.*b*_2_, *b*_1_ denotes the bias of the 2nd and 1st convolutional layers, respectively. The operator of the IN layer is set to IN. $F_{f-1}$ denotes the result of the f−1thRDB output.

After local feature fusion in each RDSB and then convolutional output, we believe that using 1×1×1 size convolutional kernels may not achieve the best results. Therefore, we use 3×3×3 convolutional kernels in our proposed RDSB. Increasing the convolutional field also avoids using too large convolutional kernels to extract too many meaningless features. To speed up the operation, we split the3×3×3 convolutional layer after local feature fusion into 3×3×1 convolutional layer and 3×1×3 convolutional layer, which is found to be more effective than the 3×3×3 convolution kernels after experiments.

Also, we found by experimental comparison that the effect of adding IN layers at different positions is not the same. Adding IN layer all after each convolutional layer is found to increase the convergence speed and higher segmentation accuracy. We will talk about it in the subsequent experimental results.

We introduce local residual learning in the RDSB to further improve the information flow, since there are multiple convolutional layers in an RDSB.

After local feature fusion in each RDSB, a jump join is performed. The feature maps after stacked convolution are summed and instance normalized with the output of $F_{f,3}$, the f−1th RDB, and passed directly to the next RDB for operation.

$F_{f,3}$ denotes the conversion layer function, which can change the convolutional layer kernel size, and different kernel sizes will play different roles. The final output of the fth RDSB is:

(4) $$F_{f}=\;IN\left[\left(F_{f,3}+F_{f,LF}+F_{f-1}\right)\right]$$

This method stacks and convolves the features of the specified layer in RDSB, and outputs them as the next input after the summation of skip connections. Efficient feature extraction and spaced jump residual concatenation are achieved.

#### 2.2.2 Leaky ReLU

It is an activation function specifically designed to solve the Dead ReLU problem, which is formulated as Leaky ReLU adjusts the zero‐gradient problem with negative values by giving a very small linear component of xto the negative input. Leak helps to extend the range of the ReLU function, usually with a value of about 0.01. In contrast to ReLU, when the network updates the iterative parameter weights, weights and biases less than 0 are not violently placed at 0, but rather the fraction less than 0 is made to exist with less impact.

#### 2.2.3 Instance normalization

BN speeds up the convergence of the model and alleviates the gradient disappearance problem. However, when the batch size is too small, the inaccuracy of the mean and variance will cause the error rate to rise, and too large a batch size will make the video memory insufficient.

IN is to find the mean and standard deviation of all pixels of a single image. The information comes from the image itself, so in a way, it can be seen as an integration and adjustment of the global information.^22^ Therefore, we try to apply it to the RDSB, while changing the position.

**FIGURE 5:**
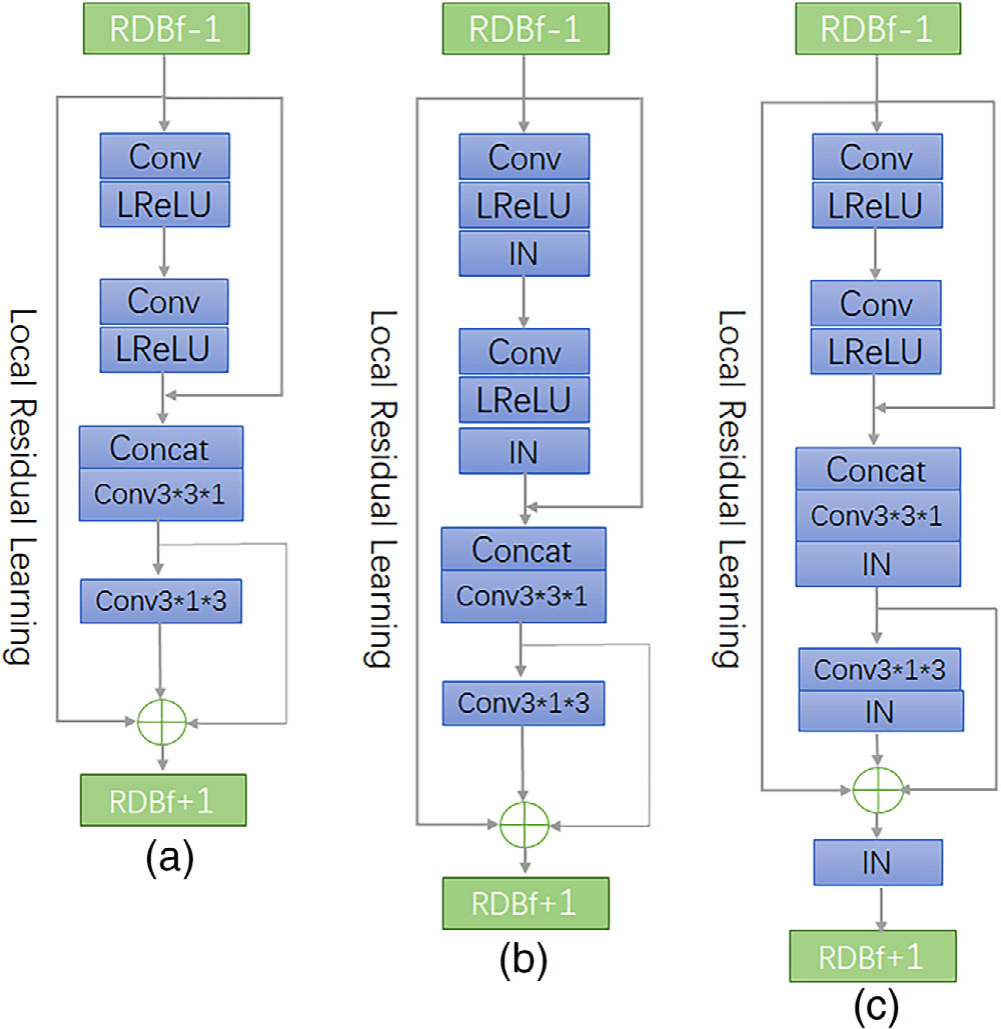
Different IN location modules (a) Module 1 (b) Module 2 (c) Module 3.

As shown in Figure 5, in order to explore the segmentation effect of IN layers in different positions, we proposed four models, the Module 1 means no IN layers, the Module 2 means before the local feature fusion, the Module 3 means after the fusion. Module 4 means the RDSB, as shown in Figure 4. The results showed that adding IN layer was better. We also found that adding the IN layer after the feature fusion in the encoder part was superior to adding it before, and finally, we tried to add the IN layer after all the convolution blocks and found the best results, which are shown in Table 1. We consider that this is brought by the excellent ability of IN to integrate the information of the picture itself.

**TABLE 1:** Quantitative results on different IN modules.

Modules	Dice	mIoU	SP	SE	HD95
Module1	78.8	66.5	99.8	82.9	22.8
Module2	74.6	63.4	99.8	77.9	24.6
Module3	79.7	67.2	99.8	82.5	16.8
Module4	82.3	70.8	99.8	86.4	9.8

### 2.3 Loss function

In upper abdominal CT images, the area occupied by the tumor is usually only about 10% of the whole image. In the segmentation task, the cross‐entropy loss function is unfriendly for data with a large amount of background like upper abdominal CT images. The dice loss function is a loss function that measures the similarity between the predicted values and the gold standard, and can optimize the contradiction between the small target and the background as well as the imbalance between positive and negative sample data.

We train our networks with a combination of dice and cross‐entropy loss:

(5) $$L_{All}=L_{Dice}\;+L_{CE}$$

The dice loss formulation used here is a multi‐class adaptation of the variant proposed in.^23^ The Dice formula is

(6) $$L_{Dice}=\;-\frac{2}{\left|N\right|}\sum_{n\varepsilon N}\frac{\sum_{i\varepsilon I}x_{i}^{n}y_{i}^{n}}{\sum_{i\varepsilon I}x_{i}^{n}+\sum_{i\varepsilon I}y_{i}^{n}}$$

wherexis the softmax output of the network andyis a one hot encoding of the groundtruth segmentation map. Both *x* and *y* have shape I×N with i∈Ibeing the number of pixels in the training patch/batch and n∈N being the classes.

Based on the hybrid loss function, we add a weight to the loss of each layer of the network for the intermediate hidden layers with low feature transparency, which decreases exponentially with resolution (divided by 2).

This allows higher resolution outputs to have more weight in the loss, solving the problem of difficulty in training shallow and intermediate networks in deep networks. This experiment is Binary classification.

Meanwhile, we replaced different hybrid loss functions to find the best results. Focal Loss and Balanced Cross Entropy Loss (BCE Loss), both of which try to solve the model training problem caused by sample imbalance. The latter adds weight factors to the loss function from the perspective of sample distribution, while the former focuses the loss on difficult samples from the perspective of sample classification ease. We will mention the effects produced by the different hybrid loss functions in the subsequent Table 3.

**TABLE 2:** Quantitative results on different mixed loss functions.

Different mixed loss functions	Dice	mIoU	SP	SE	HD95
Focal Loss +CE Loss	73.3	61.5	99.9	73.4	17.4
Dice Loss + Focal Loss	81	69.3	99.8	84.5	9.8
Dice Loss + BCE Loss	82.2	70.7	99.8	86.2	9.8

### 2.4 Experiment environment

We implemented the network using Pytorch. The computer operating system is Ubuntu 18. 04, and a GPU is used to accelerate the training and testing of the network model, with a graphics card model Nvidia GTX 1080Ti. The Adam optimizer is used because the Adam algorithm can adjust the learning rate adaptively during training and has a faster convergence rate.

Increasing the batch size will speed up the process, but in disguise, more Epochs are needed to achieve the required accuracy. At the same time, as the number of epochs increases, the number of updates of the weights in the neural network also increases, and the model becomes overfitted from underfitting. To prevent the segmentation effect from deteriorating after overfitting, we set the batch size to 2, the learning rate to 1e‐3, the epoch to 100, and the training iterations to 250 based on our experience and the results of changing the hyperparameter training several times. The model converges smoothly and has the best effect.

The inference is performed using a five‐fold cross‐validation of the resulting five trained model integration, and to increase the stability of the network, the patches are sampled in such a way that More than 1/3 of the pixels in a batch sample are pixels of the foreground class.

### 2.5 Dataset and evaluation metrics

**FIGURE 6:**
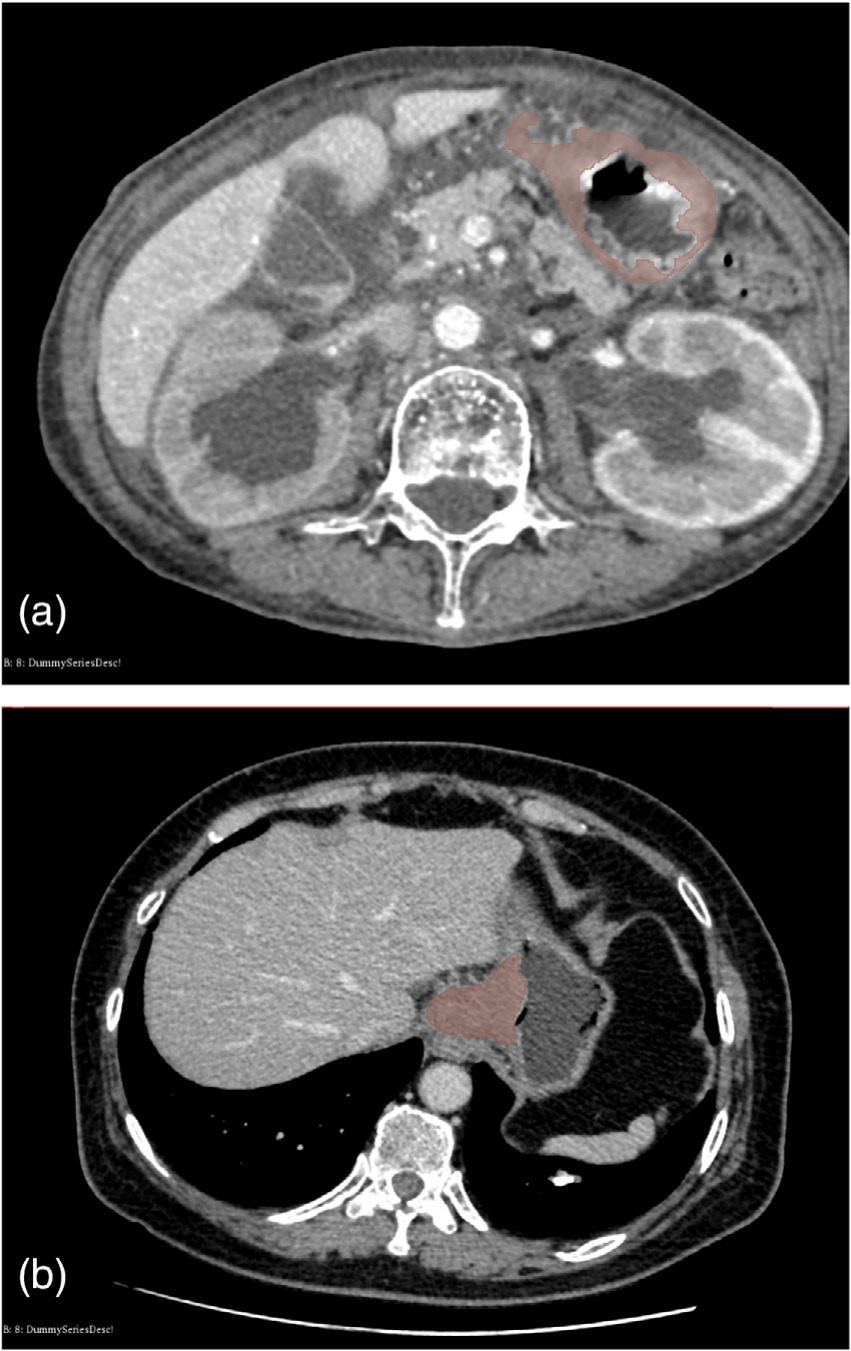
Different CT sequence diagrams of the different patients (a) case 1 (b) case 2.

The dataset uses the data of gastric adenocarcinoma provided by a hospital and annotated by a university researcher, as shown in Figure 6. The red part is the lesion area. 134 cases of experimental data are used, each group of CT sequences contains the number of slices of gastric adenocarcinoma ranging from 10 to 68 in size, and the CT image size is initially 360 × 360 mm.

We combined the number of slices in dicom format for each group of CT sequences into a complete nii format and put them into the network for training. The data are cropped, resampled, pixel normalized, etc., while data enhancements such as random rotation, on‐the‐fly scaling, random elastic transformation, gamma correction, mirroring, etc. are performed.

The purpose of normalization is to make the grayscale values of each image in the training set have the same distribution. CT images do normalization with the mean and standard deviation of the foreground of the whole training set. In CT images, the intensity information HU values can reflect the physical properties of different tissues, and using the statistical information of the foreground of the whole training set can effectively use the extra information of HU values.

The second point is that there are often unusually large isolated values and unusually small isolated values in CT images, and it is necessary to first clip the image HU values to between [0.5, 99.5] percentage range of foreground HU values. The data are divided into a training set of 108 cases and a test set of 26 cases for five‐fold cross‐validation.

This study uses Dice, Mean Intersection Over Union (mIoU), and 95% Hausdorff distance(HD95) as the main evaluation. Dice and mIoU values indicate better performance of the algorithm. The larger the value of Dice and mIoU, the better the performance of the algorithm, and the smaller the Hausdorff distance 95, the higher the accuracy of edge segmentation.

## 3 RESULTS AND DISCUSSION

To demonstrate the effectiveness of the proposed method, this paper was compared with TransUNet,^24^ HiFormer,^25^ nnUNet,^13^ and nnFormer^26^ multiple segmentation networks for the same segmentation task. The network segmentation results using the CT image data of gastric adenocarcinoma outlined by physicians are shown in Table 1. This method performs well in most indexes and outperforms the segmentation results of other networks in three indexes, Dice coefficient, mIoU and HD95. SP, SE are specificity and sensitivity.

The Dice coefficient is an ensemble similarity measure function that is commonly used to calculate the similarity of two samples. However, it is insensitive to boundaries and only considers the degree of overlap between the two sets without taking into account the spatial relationship between pixels. MIoU is the overlap area between predicted segmentation and labels divided by the joint area between predicted segmentation and labels. It has low sensitivity to small targets and is not sensitive to boundaries, but takes into account pixel spatial relationships. As with Dice, the advantages are simplicity and intuition, not affected by sample imbalance and better robustness.

Hausdorff Distance 95 is used as the main segmentation metric because it can measure the segmentation accuracy of gastric adenocarcinoma boundaries in CT images, and also to verify whether the proposed method can reduce the occurrence of mis‐segmentation of gastric adenocarcinoma boundaries. Dice is more sensitive to the internal padding of the mask, while Hausdorff Distance is more sensitive to the segmented boundaries. Hausdorff Distance 95 is the final value multiplied by 95%, in order to eliminate unreasonable distances caused by some outliers, maintain overall numerical stability, and be more effective in boundary segmentation.

**FIGURE 7:**
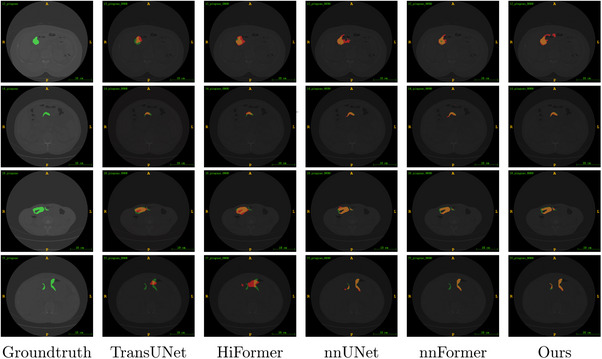
Visual comparison of slice segmentation results from different methods.

**FIGURE 8:**
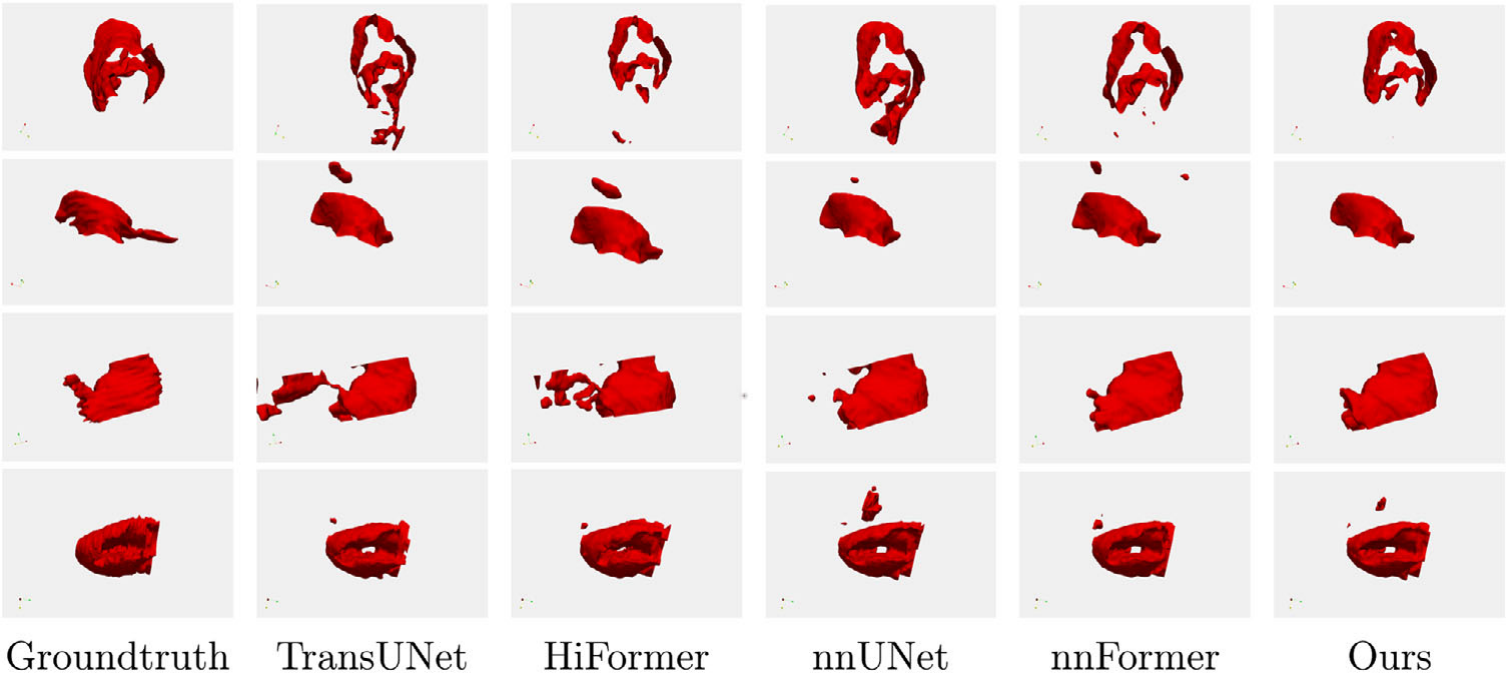
Visual comparison of 3D segmentation results from different methods.

As shown in Figure 7, we selected slice images from different cases in the dataset to visualize the segmentation results of the five models. The first column of each row in Figure 7 outlines the image as ground truth, the middle four columns show the segmentation results of different networks, and the last column shows our method. The green marker is the label, the red marker is the segmentation result, and the orange color shows the part where the label intersects the result. The 3D display images of some cases are shown in Figure 8.

From Figure 6, it can be seen that there are uneven contrast, blurred edges, different morphological sizes, and artifacts in the CT images of upper abdomen of different patients. Markers are required to manually adjust the window width and window position before marking to make the image clearer and thus easier to mark. It also increases the task volume.

TransUNet, HiFormer both combine CNN and Transformer structures and focus on local detail information and global information extraction. However, HiFormer focuses on the jump connection in the encoder‐decoder structure by proposing a two‐stage fusion module. As with TransUNet, the morphology and size of gastric lesions are poorly perceived, as are the presence of artifacts. For small targets and the emergence of multiple disease areas segmentation effect is not good. Thus, the image segmentation results are not well suited to the physician's analysis needs.

Table 1 HD metrics show that our proposed method is more accurate for image edge segmentation. Our method focuses on feature fusion in the encoder part, improves feature reuse by residual dense skip connections, changes the size of the convolutional layers to avoid extracting too many meaningless features, and the addition of the IN layer allows the image to integrate information about its own pixels. Meanwhile, the hybrid loss function of Dice combined with CE Loss optimizes the imbalance between target and background and handles positive and negative samples fairly.

Table 2 shows the results of the IN layer at different positions. It can be seen that the results after the IN layer is placed on the local feature fusion are much better than before, and we think it is due to the fact that the local feature fusion preserves the accumulated features adaptively while increasing the perceptual field after changing the convolution size.

**TABLE 3:** Quantitative results on different methods.

Methods	Dice	mIoU	SP	SE	HD95
TransUNet[27]	77	68.4	80.6	74.1	26.2
HiFormer[28]	78.5	69.8	80.9	80.9	25.6
nnFormer[29]	81.6	69.9	99.9	81.6	15
nnUNet[21]	81.4	69.5	99.8	85.9	31.4
Ours	82.3	70.8	99.8	86.4	9.8

Also by applying all IN layers on RDSB, IN layers calculate the mean and variance by channel‐by‐channel (for each feature map), the image itself has more complete pixel information integration and better segmentation.

Table 3 shows the results of different hybrid loss functions, and compared with Table 1 Ours method, it is still the hybrid loss function of Dice Loss combined with CE Loss that has the most superior effect. We know that all four loss functions are suitable for small target segmentation network training.

Focal Loss, based on BCE Loss, adds a modulation factor to reduce the weight of easy‐to‐score samples, focuses on the training of difficult samples, and is easily disturbed by noise.

The CE Loss requires the model to be very confident in its prediction, so the generalization ability of the model will be reduced, and it is not effective to use in the presence of a large amount of background. From Table 3, it can be seen that the combination of the two has the worst segmentation effect.

Dice loss can alleviate the negative impact of foreground‐background imbalance in the sample, and its training is more concerned with mining the foreground region, but there will be a loss saturation problem. CE Loss is calculated equally for each pixel. The combination of Dice loss and BCE loss can improve the stability of model training and the segmentation effect.

Table 4 shows the ablation experiment, where Baseline is the basic 3D‐UNet in the proposed network structure. It can be seen that the segmentation results with the addition of RB and RDB structures have improved compared to Baseline, indicating that using a super‐resolution module for CT image data preprocessing to improve the segmentation effect is the correct direction.

**TABLE 4:** Ablation study.

Methods	Dice	mIoU	SP	SE	HD95
Baseline	74.6	63.4	77.9	99.8	24.6
Baseline+RB	78.8	66.5	82.9	99.8	22.8
Baseline+RDB	80.5	68.6	84.7	99.8	23.95

Despite the good results obtained, these results may only be applicable to hospital‐based cancer datasets. The extreme imbalance of medical CT images itself, the large number of normal samples and easy accessibility, the small number of lesion samples and their complexity and variability, as well as the presence of noise pollution and artifacts, lead to the fact that existing medical image segmentation models still suffer from scale limitations and insufficient generalization ability, which limit the performance and applicability of the models in practical applications.

However, we consider that the proposed RDSB module focuses on the preprocessing of CT image datasets to further capture features and enhance segmentation performance by improving the data image quality and preserving image details and edge information. It incorporates the RDB module for removing CT image artifacts in super‐resolution, dense connectivity improves the backpropagation of gradients for effective feature fusion and interval jump connectivity, and feature map stacked convolution improves the reusability of features. By replacing the BN layer with an IN layer and modifying the position of the IN layer, the image global information is integrated and adjusted once. There is reason to believe that there is potential applicability in certain cancer datasets with blurred and unclear surrounding tissue boundaries and artifact problems, which requires further research to improve the breadth and accuracy of its application in clinical diagnosis.

Meanwhile, there are still some limitations, and the performance advantage of 3D model is not obvious for some thick CT image data with poor interlayer continuity. Replacing the convolutional kernel size has limited effect even though it increases the receptive field, and it is still difficult to model the explicit long‐distance dependence. This inherent limitation of convolutional operations can prevent CNNs from learning global semantic information, but this ability is crucial for intensive prediction tasks such as segmentation. By utilizing the existing super‐resolution technology module RDB on natural images and applying it to CT medical images, the medical imaging principles between different modalities are different, which can result in different corresponding relationships between sample pairs and may have a certain impact on segmentation. At the same time, different network channels and training methods and other hyperparameters are also used, the experimental setup chosen may not necessarily be the optimal choice for different tasks.

## 4 CONCLUSION

We presented an end‐to‐end 3D medical image segmentation model architecture based on a residual dense jump structure, as well as exploring experiments with different hybrid loss functions, which provides accurate lesion segmentation for CT images of gastric adenocarcinoma in the upper abdomen. In our setup of five‐fold cross‐validation experiments, the model incorporating the hybrid loss function of Dice and CE obtained a Dice coefficient of 82.3% with an average cross‐merge ratio of 70.8%. Compared with other methods applied to CT images of gastric adenocarcinoma, our method has some advantages. However, the segmentation results are still lacking due to the insufficient size and quality of the dataset as well as the limitations of the deep learning network model. Further research is needed to improve the model generalization.

## AUTHOR CONTRIBUTIONS

Ying Hu wrote the original draft, data collection and analysis, and innovative design of research methods. Yue Guo and Xian Xu assisted in data processing and paper discussion and revision. Shipeng Xie provided experimental equipment and supervised and coordinated the entire research planning.

## CONFLICT OF INTEREST STATEMENT

The authors declare no conflicts of interest.

## Data Availability

The data that support the findings of this study are available on request from the corresponding author. The data are not publicly available due to privacy or ethical restrictions.
